# Association Between Coronavirus Disease 2019 and Acute Complicated Diverticulitis

**DOI:** 10.7759/cureus.33252

**Published:** 2023-01-02

**Authors:** Christian Karime, Paul Travers, Ahmed Ouni, Dawn Francis

**Affiliations:** 1 Department of Internal Medicine, Mayo Clinic, Jacksonville, USA; 2 Department of Gastroenterology and Hepatology, Mayo Clinic, Jacksonville, USA

**Keywords:** covid-19, coronavirus disease 2019 (covid-19), hospital internal medicine, acute diverticulitis, intestinal perforation, complicated diverticulitis

## Abstract

Background

Gastrointestinal manifestations of coronavirus disease 2019 (COVID-19) are increasingly recognized. Through potentially overlapping pathophysiology, co-occurrence of COVID-19 and first-time acute diverticulitis has been reported. Our study aims to further characterize this association in COVID-19-positive patients within a large tertiary care academic center.

Methodology

Patients diagnosed with COVID-19 who subsequently developed acute diverticulitis within 30 days were identified between 2020 and 2022. COVID-19 and acute diverticulitis were diagnosed by polymerase chain reaction and computed tomography, respectively. Patients with prior history of acute diverticulitis were excluded. Patient characteristics and comorbid conditions were collected. Characterization of the COVID-19 course (treatment setting, medical/ventilatory therapy) and acute diverticulitis (treatment setting, medical/surgical therapy, complications) was performed retrospectively. Subanalysis was performed by COVID-19 vaccination status, the severity of COVID-19, and the timing of acute diverticulitis diagnosis.

Results

A total of 81 patients were identified, with a median duration between COVID-19 diagnosis and acute diverticulitis of 13 days (interquartile range = 2.5-21.0), with 44.4% of patients requiring hospitalization for COVID-19. The all-cause complication rate of acute diverticulitis was noted to be 59.3%, most commonly intestinal perforation (39.5%), abscess formation (37.0%), and peritonitis (14.8%). Although a trend toward increased all-cause complications (65.9%), intestinal perforation (43.9%), and peritonitis (19.5%) was noted in unvaccinated patients, this did not reach significance. Although all-cause complication rate did not differ in patients diagnosed with acute diverticulitis at the time of COVID-19 presentation, a significantly elevated incidence of intestinal perforation (55.9% vs. 27.7%, p = 0.01), peritonitis (29.4% vs. 4.3%, p < 0.01), and the need for emergent surgical intervention (38.2% vs. 10.6%, p < 0.01) was noted.

Conclusions

Our study indicates that patients diagnosed with first-time acute diverticulitis within 30 days of COVID-19 infection have a high complication rate, most commonly intestinal perforation. Additionally, patients diagnosed with acute diverticulitis at the same time as COVID-19 detection had a significantly elevated rate of complications and emergent surgical needs. Given the high complication rate, patients who develop diverticulitis within a short timeframe of COVID-19 infection may benefit from increased clinician vigilance and monitoring.

## Introduction

The coronavirus disease 2019 (COVID-19) pandemic has been at the forefront of public health concern, with over 90 million cases reported to date in the United States. Presenting primarily with fever and respiratory tract symptoms, extrapulmonary manifestations are increasingly being recognized as part of the COVID-19 disease spectrum. Gastrointestinal manifestations occur in a significant proportion of patients with COVID-19, with the most common symptoms being nausea, vomiting, diarrhea, anorexia, and abdominal pain [1-3]. While incompletely understood, the multifactorial pathophysiology of gastrointestinal injury in COVID-19 is thought to include a combination of direct virus-mediated tissue damage, diffuse endothelial and submucosal vascular inflammation, intestinal edema, and virus-mediated alterations to the intestinal microbiome [2,4,5]. As such, there are potentially several mechanisms by which COVID-19 may result in gastrointestinal sequela of infection.

Acute diverticulitis is postulated to occur through the obstruction of intestinal diverticula by fecal matter, resulting in low-grade inflammation and mucosal abrasion that eventually lead to bacterial overgrowth and intestinal microperforation [6]. Recent studies have shown an association between chronic inflammatory states, altered gut microbiome, and the development of acute diverticulitis [7,8]. With potentially overlapping pathophysiologic mechanisms, an association between COVID-19 infection and acute diverticulitis has been reported [9-11]. However, together representing less than a handful of patients, this association has not been investigated on a larger scale. In the current work, we aimed to investigate the association between COVID-19 infection and the first occurrence of acute diverticulitis in patients with COVID-19 infection. Given the reduction of severe COVID-19 infection in patients vaccinated against COVID-19 [12], vaccinated and unvaccinated patients were compared. Given both direct and inflammation-induced gastrointestinal injury in COVID-19 infection, the authors hypothesize that patients who develop acute diverticulitis shortly after COVID-19 infection may have a more complicated disease course with an elevated risk of complications. Furthermore, given that inflammation is more severe in active COVID-19 infection, we hypothesize that patients who develop acute diverticulitis shortly after COVID-19 infection would have an increased incidence of complications.

## Materials and methods

Patient selection

Institutional Review Board approval was obtained to collect de-identified patient information through a retrospective chart review at a large tertiary care academic center in the United States (Mayo Clinic Institutional Review Board, approval number: 21-005645). Patients were selected by filtering International Classification of Diseases 10 (ICD10) codes for confirmed positive COVID-19 diagnosis (U07.1) and cross-references with ICD10 codes for the diagnosis of acute diverticulitis within the subsequent 30 days (K57.*) to obtain the initial patient cohort. The time frame of the retrospective review was January 1, 2020, to August 30, 2022. The diagnosis of COVID-19, COVID-19 vaccination status, and acute diverticulitis was then confirmed through a detailed retrospective chart review. A confirmed positive COVID-19 infection was defined as a positive reverse transcription polymerase chain reaction (RT-PCR) assay on samples taken from the patients’ nasopharynx. The diagnosis of acute diverticulitis was defined based on previously reported computed tomography (CT) imaging criteria. Specifically, the diagnostic criteria used included imaging findings of colonic wall thickening (wall thickness greater than 3 mm on luminal short axis) and peri-colonic fat stranding [13]. Patients were excluded if a diagnosis of COVID-19 or acute diverticulitis could not be confirmed. Patients with a prior history of acute diverticulitis were excluded. Patients whose diagnosis of acute diverticulitis occurred more than 30 days after the initial positive COVID-19 diagnosis were also excluded.

Data collection

Clinical, laboratory, and imaging data were obtained through a retrospective review. The following demographic data were collected: age, gender, ethnicity, and body mass index (BMI). Clinical variables included common comorbid conditions (diabetes mellitus, hypertension, hyperlipidemia, obesity, chronic obstructive pulmonary disease (COPD), obstructive sleep apnea, and diverticulosis). Current or previous alcohol and tobacco history were also collected. COVID-19-related variables were collected, including the date of diagnosis, vaccination status at the time of diagnosis, COVID-19-directed medical therapy, COVID-19-related hospitalization and length of stay, need for intubation, mechanical ventilation duration, use of non-invasive positive pressure ventilation (NIPPV), and use of high-flow oxygen supplementation. Data regarding acute diverticulitis was also collected, including date of acute diverticulitis diagnosis, treatment setting (outpatient vs. inpatient), hospital length of stay, and complications related to acute diverticulitis (abscess formation, perforation defined by abdominal free air, fistula formation, peritonitis, and the need for emergent surgical intervention).

Statistical analysis

Statistical analysis was performed using SPSS statistical software version 25.0 (IBM Corp., Armonk, NY, USA). The data were summarized using descriptive statistics. Values are reported as the median and interquartile range (IQR) or fractions and percentages, as appropriate. Wilcoxon rank sum and Fisher’s exact tests were used to compare baseline characteristics and incidence of acute diverticulitis complications between groups.

## Results

Demographic and clinical characteristics

A retrospective review identified 81 patients with confirmed COVID-19 infection who were subsequently diagnosed with acute diverticulitis within 30 days. Baseline characteristics are summarized in Table 1. Of the 81 patients included in the final analysis, 40 (49.4%) were male, with a median age of 67 years (IQR = 57.5-76.5). In total, 69 (85.2%) patients identified as Caucasian, with six (7.4%) identifying as Hispanic, and three (3.7%) identifying as African American. The median BMI was 29.2 (IQR = 25.8-34.1). Of the 81 patients, the following comorbidities were noted: 50 (61.7%) had hypertension, 41 (50.6%) had hyperlipidemia, 17 (21.0%) had diabetes mellitus, 20 (24.7%) had COPD, and 16 (19.8%) had obstructive sleep apnea. Regarding previously diagnosed gastrointestinal disorders, 32 (39.5%) patients had known diverticulosis. No significant differences were noted between vaccinated and unvaccinated patients or COVID-19 hospitalized versus non-hospitalized patients on the above baseline demographics and comorbid conditions (Table 1). Similarly, no significant differences were noted between patients diagnosed with acute diverticulitis at the time of COVID-19 detection versus after COVID-19 detection (Table 2).

**Table 1:** Baseline characteristics and incidence of acute diverticulitis complications. Baseline patient characteristics, COVID-19-associated variables, and incidence rates of acute complicated diverticulitis are detailed. Patients were divided into those who were vaccinated versus those not vaccinated for COVID-19 at the time of acute diverticulitis presentation as well as by COVID-19 hospitalization status. Wilcoxon rank sum and Fisher’s exact tests were used for statistical analysis. No significant differences were noted between vaccinated and unvaccinated patients on demographic as well as comorbid conditions. Similarly, no significant differences were noted in patients hospitalized versus those not hospitalized for COVID-19. Regarding complications of acute diverticulitis, the overall rate of all-cause complications was similar between vaccinated and unvaccinated patients. Although a higher incidence of perforation, peritonitis, and the need for emergent surgery was noted in unvaccinated patients, which did not reach significance. For patients who required hospitalization for COVID-19, the incidence of peritonitis and the need for emergent surgery was significantly elevated. COVID-19 hospitalized patients also had a non-significant trend toward a higher incidence of all complications, abscess formation, perforation, and fistula formation. *: p < 0.05. IQR = interquartile range; BMI = body mass index; COVID-19 = coronavirus disease 2019

	Median (IQR) or fraction (%)						
Variable	All patients (N =81)	Vaccinated (N = 40)	Unvaccinated (N = 41)	P-value	Hospitalized (N = 36)	Not hospitalized (N = 45)	P-value
Demographics							
Age (years)	67 (57.5-76.5)	70 (60.3-81)	62 (52.5-74.0)	0.17	71 (60.3-80.3)	62 (54.0-74.5)	0.40
Male	40 (49.4%)	21 (52.5%)	19 (46.3%)	0.66	20 (55.6%)	20 (44.5%)	0.38
BMI	29.2 (25.8-34.1)	28.4 (25.1-33.6)	29.7 (27.5-34.2)	1.00	28.5 (25.0-34.1)	29.7 (26.1-34.6)	1.00
Caucasian	69 (85.2%)	37 (92.5%)	32 (78.0%)	0.12	30 (83.3%)	39 (86.7%)	0.76
Hispanic	6 (7.4%)	2 (5.0%)	4 (9.8%)	-	1 (2.8%)	5 (11.1%)	-
African American	3 (3.7%)	1 (2.5%)	2 (4.9%)	-	2 (5.6%)	1 (2.2%)	-
Comorbidities							
Obesity (>30 BMI)	36 (44.4%)	17 (42.5%)	19 (46.3%)	0.82	14 (38.9%)	22 (48.9%)	0.50
Diabetes mellitus	17 (21.0%)	5 (12.5%)	12 (29.3%)	0.10	10 (27.8%)	7 (15.6%)	0.27
Hypertension	50 (61.7%)	25 (62.5%)	25 (61.0%)	1.00	23 (63.9%)	27 (60.0%)	0.82
Hyperlipidemia	41 (50.6%)	20 (50.0%)	21 (51.2%)	1.00	20 (55.6%)	21 (46.7%)	0.51
Obstructive sleep apnea	16 (19.8%)	12 (30%)	8 (19.5%)	0.31	6 (16.7%)	14 (31.1%)	0.20
Chronic obstructive pulmonary disease	20 (24.7%)	11 (27.5%)	5 (12.2%)	0.10	10 (27.8%)	6 (13.3%)	0.16
Diverticulosis	32 (39.5%)	18 (45.0%)	14 (34.1%)	0.38	13 (36.1%)	19 (42.2%)	0.65
Tobacco use	40 (49.4%)	24 (60%)	16 (39.0%)	0.08	16 (44.4%)	24 (53.3%)	0.51
Former	33 (40.7%)	20 (50.0%)	13 (31.7%)	-	13 (36.1%)	20 (44.4%)	-
Current	7 (8.6%)	4 (10.0%)	3 (7.3%)	-	3 (8.3%)	4 (8.9%)	-
Alcohol use	53 (65.4%)	26 (65.0%)	27 (65.9%)	1.00	24 (66.7%)	29 (64.4)	1.00
Former	23 (28.4%)	14 (35.0%)	9 (22.0%)	-	11 (30.6%)	12 (26.7%)	-
Current	30 (37.0%)	12 (30.0%)	18 (43.9%)	-	13 (36.1%)	17 (37.8%)	-
COVID-19-associated variables							
Hospitalization for COVID-19	36 (44.4%)	13 (32.5%)	23 (56.1%)	0.04*	-	-	-
Hospitalization duration (days)	10.5 (6-16)	10 (6.5-16.5)	12 (5.0-16.0)	0.43	10.5 (6-16)	-	-
Intubation	4 (4.9%)	0	4 (9.8%)	0.12	4 (11.1%)	-	-
Non-invasive positive pressure ventilation	13 (16.0%)	3 (7.5%)	10 (24.4%)	0.07	13 (36.1%)	-	-
High-flow nasal cannula	16 (19.8%)	3 (7.5%)	13 (31.7%)	0.01*	16 (44.4%)	-	-
Acute diverticulitis-associated variables							
The time between the diagnosis of COVID-19 and diverticulitis (days)	13 (2.5-21.0)	8 (0.0-21.5)	14 (5.0-21.0)	0.03*	7 (1.25-19.8)	15 (3.5-23.5)	0.61
Diverticulitis diagnosed at the time of COVID-19 detection	33 (40.7%)	17 (42.5%)	16 (39.0%)	0.82	25 (69.4%)	8 (17.8%)	<0.01*
Hospitalization for diverticulitis	56 (69.1%)	27 (67.5%)	29 (70.7%)	0.81	33 (91.7%)	23 (51.1%)	NA
Acute diverticulitis complications							
Any complication	48 (59.3%)	21 (52.5%)	27 (65.9%)	0.26	34 (66.7%)	24 (53.5%)	0.26
Abscess formation	30 (37.0%)	15 (37.5%)	15 (36.6%)	1.00	14 (38.9%)	16 (35.6%)	0.82
Intestinal perforation	32 (39.5%)	14 (35.0%)	18 (43.9%)	0.50	18 (50.0%)	14 (31.1%)	0.11
Peritonitis	12 (14.8%)	4 (10.0%)	8 (19.5%)	0.35	10 (27.8%)	2 (4.4%)	<0.01*
Fistula formation	4 (4.9%)	2 (5.0%)	2 (4.9%)	1.00	3 (8.3%)	1 (2.2%)	0.32
Emergent surgery needs	18 (22.2%)	8 (20.0%)	10 (24.4%)	0.79	13 (36.1%)	5 (11.1%)	<0.01*

**Table 2:** Baseline characteristics and incidence of acute diverticulitis complications – subanalysis by the time of acute diverticulitis diagnosis. Baseline patient characteristics, COVID-19-associated variables, and incidence rates of acute complicated diverticulitis are detailed. Patients were divided into those diagnosed with acute diverticulitis at the time versus those after the detection of COVID-19. Wilcoxon rank sum and Fisher’s exact tests were used for statistical analysis. No significant differences were noted in demographic and comorbid conditions. Regarding complications of acute diverticulitis, the overall rate of all-cause complications was similar between patients diagnosed with acute diverticulitis at the time versus after the detection of COVID-19. The incidence of intestinal perforation, peritonitis, and the need for emergent surgery was significantly elevated in patients diagnosed with acute diverticulitis at the time of COVID-19 detection. Although a higher incidence of abscess and fistula formation was noted, this difference did not reach significance. *: p < 0.05. IQR = interquartile range; BMI = body mass index; COVID-19 = coronavirus disease 2019

	Median (IQR) or fraction (%)			
Variable	All patients (N = 81)	Diverticulitis at COVID-19 diagnosis (N = 34)	Diverticulitis within 30 days of COVID-19 diagnosis (N = 47	P-value
Demographics				
Age (years)	67.0 (57.5-76.5)	68.5 (59.0-75.3)	67.0 (54.0-79.0)	0.27
Male	40 (49.4%)	17 (50.0%)	23 (48.9%)	1.00
BMI	29.2 (25.8-34.1)	29.3 (25.0-33.5)	29.0 (26.3-34.4)	1.00
Caucasian	69 (85.2%)	27 (79.4%)	42 (89.4%)	0.34
Hispanic	6 (7.4%)	2 (5.9%)	4 (8.5%)	-
African American	3 (3.7%)	2 (5.9%)	1 (2.1%)	-
Comorbidities				
Obesity (>30 BMI)	36 (44.4%)	14 (41.2%)	22 (46.8%)	0.66
Diabetes mellitus	17 (21.0%)	7 (20.6%)	10 (21.3%)	1.00
Hypertension	50 (61.7%)	25 (73.5%)	25 (53.2%)	0.07
Hyperlipidemia	41 (50.6%)	19 (55.9%)	22 (46.8%)	0.50
Obstructive sleep apnea	16 (19.8%)	6 (17.6%)	14 (29.8%)	0.30
Chronic obstructive pulmonary disease	20 (24.7%)	8 (23.5%)	8 (17.0%)	0.57
Diverticulosis	32 (39.5%)	10 (29.4%)	22 (46.8%)	0.17
Tobacco use	40 (49.4%)	16 (47.1%)	24 (51.1%)	0.82
Former	33 (40.7%)	11 (32.4%)	22 46.8%)	-
Current	7 (8.6%)	5 (14.7%)	2 (4.3%)	-
Alcohol use	53 (65.4%)	22 (64.7%)	31 (66.0%)	1.00
Former	23 (28.4%)	8 (23.5%)	15 (31.9%)	-
Current	30 (37.0%)	14 (41.2%)	16 (34.0%)	-
COVID-19-associated variables				
Hospitalization for COVID-19	36 (44.4%)	25 (73.5%)	11 (23.4%)	<0.01*
Hospitalization duration (days)	10.5 (6-16)	9.5 (6.3-29.3)	15.0 (8.5-19.0)	0.02*
Intubation	4 (4.9%)	4 (11.8%)	0	0.03*
Non-invasive positive pressure ventilation	13 (16.0%)	9 (26.5%)	4 (8.5%)	0.04*
High-flow nasal cannula	16 (19.8%)	13 (38.2%)	3 (6.4%)	<0.01*
Acute diverticulitis-associated variables				
The time between the diagnosis of COVID-19 and diverticulitis (days)	13 (2.5-21.0)	0	1.0 (0.0-6.0)	-
Diverticulitis diagnosed at the time of COVID-19 detection	33 (40.7%)	34 (100%)	0	-
Hospitalization for diverticulitis	56 (69.1%)	29 (85.3%)	27 (57.4%)	<0.01*
Acute diverticulitis complications				
Any complication	48 (59.3%)	23 (67.6%)	25 (53.2%)	0.25
Abscess formation	30 (37.0%)	13 (38.2%)	17 (36.2%)	1.00
Intestinal perforation	32 (39.5%)	19 (55.9%)	13 (27.7%)	0.01*
Peritonitis	12 (14.8)	10 (29.4%)	2 (4.3%)	<0.01*
Fistula formation	4 (4.9%)	2 (5.9%)	2 (4.3%)	1.00
Emergent surgery needs	18 (22.2%)	13 (38.2%)	5 (10.6%)	<0.01*

Of the 81 patients, 53 (65.4%) had a history of current or former alcohol use, with 30 (37.0%) currently consuming more than two alcoholic beverages per week at the time of COVID-19 diagnosis. Regarding tobacco usage, 40 (49.4%) patients had a history of current or former tobacco usage, with seven (8.6%) currently using tobacco products at the time of COVID-19 diagnosis. No significant differences were noted between vaccinated and unvaccinated patients or COVID-19 hospitalized versus non-hospitalized patients were noted (Table 1). Similarly, no significant differences were noted between patients diagnosed with acute diverticulitis at the time of COVID-19 detection versus after COVID-19 detection (Table 2).

COVID-19-related data

Of the 81 patients, 36 (44.4%) patients required hospital treatment for COVID-19 due to the severity of symptoms (median duration = 10.5 days, IQR = 6-16 days). A significant difference was noted in the rate of hospitalization for COVID-19 between vaccinated and unvaccinated patients (32.5% vs. 56.1%, p < 0.05). Although median hospitalization duration differed between vaccinated and unvaccinated patients, this did not reach significance (10 vs. 12 days, p = 0.43). Of the 36 hospitalized patients, 33 (40.7%) required oxygen supplementation beyond that available by a nasal cannula. This included four (4.9%) patients requiring intubation (median duration = 9.5 days, IQR = 6.3-29.3 days), 13 (16.0%) requiring NIPPV, and 16 (19.8%) requiring high-flow nasal cannula. For unvaccinated patients, the most used COVID-19-directed medical therapy was remdesivir (21/41 patients, 51.2%), dexamethasone (21/41 patients, 51.2%), azithromycin (4/41 patients, 9.8%), convalescent plasma infusion (2/41 patients, 4.9%), tocilizumab (2/41 patients, 4.9%), and oral prednisone (2/41 patients, 4.9%). In total, 18 (43.9%) patients received no therapy for COVID-19. For vaccinated patients, the most used COVID-19-directed medical therapy was remdesivir (11/40 patients, 27.5%), dexamethasone (8/40 patients, 20%), convalescent plasma infusion (4/40 patients, 10%), nirmatrelvir/ritonavir (3/40 patients, 7.5%), azithromycin (2/40 patients, 5%). Seventeen (41.5%) patients received no therapy for COVID-19.

Acute diverticulitis

The median duration between the diagnosis of COVID-19 and acute diverticulitis was 13 days (IQR = 2.5-21.0 days), with 33 (40.7%) patients being diagnosed with acute diverticulitis at the time of COVID-19 detection. Comparing vaccinated and unvaccinated patients, vaccinated patients had an overall shorter median time between COVID-19 diagnosis and diagnosis of acute diverticulitis (8 vs. 14 days, p < 0.05). However, this is of unclear clinical significance. No difference was noted in the proportion of patients who were diagnosed with acute diverticulitis at the time of COVID-19 diagnosis (42.5% vs. 39.0%, p = 0.82). Comparing patients hospitalized for COVID-19 versus patients not requiring hospitalization, no difference was noted in median time between COVID-19 diagnosis and acute diverticulitis diagnosis (p = 0.61). Of the patients hospitalized for COVID-19 who later developed acute diverticulitis, 69.4% were diagnosed with acute diverticulitis upon initial presentation to the hospital (Table 1). This was significantly higher compared to non-hospitalized patients (69.4% vs. 17.8%, p < 0.01).

Of the 81 patients diagnosed with acute diverticulitis following COVID-19 diagnosis, 56 (69.1%) required hospital-level treatment for diverticulitis while the remaining 25 (30.9%) were managed in the outpatient setting. No difference was noted between vaccinated and unvaccinated patients (67.5% vs. 70.7%, p = 0.81). For patients hospitalized for COVID-19, 33 of 36 (91.7%) patients were diagnosed with acute diverticulitis while hospitalized. For patients not requiring hospitalization for COVID-19, 23 (51.1%) required in-hospital treatment for acute diverticulitis.

Acute diverticulitis and the incidence of diverticular complications

Overall Complication Rate

As detailed in Table 1, acute complicated diverticulitis (at least one complication) occurred in 59.3% (48/81) of patients. Intestinal perforation, defined by the presence of intra-abdominal free air, was noted to be the most common complication and occurred in 39.5% (32/81) of patients. Overall, 37.0% (30/81) of patients were noted to have abscess formation, 14.8% (12/81) were noted to have peritonitis, and 4.9% (4/81) were noted to have fistula formation. Of the 81 patients, 18 (22.2%) patients underwent emergent surgical intervention.

Comparison of Complication Rate by COVID-19 Vaccination Status

Comparing COVID-19 vaccinated versus unvaccinated patients who developed acute diverticulitis, unvaccinated patients had a trend toward a higher incidence of acute complicated diverticulitis (65.9% vs. 52.5%). However, this did not reach significance (p = 0.26). A higher rate of perforation (43.9% vs. 35.0%, p = 0.50), peritonitis (19.5% vs. 10.0%, p = 0.35), and need for emergent surgery (24.4% vs. 20.0%, p = 0.79) was noted in unvaccinated patients; however, these did not reach significance. A similar rate of abscess formation (36.6% vs. 37.5%, p = 1.00) and fistula formation (4.9% vs. 5.0%, p = 1.00) was noted. Detailed results are available in Table 1.

Comparison of Complication Rate by COVID-19 Hospitalization Status

Patients with COVID-19 who required hospitalization for treatment had a trend toward a higher incidence of acute complicated diverticulitis (66.7% vs. 53.5%). However, this did not reach significance (p = 0.26). Regarding individual complications, a significantly higher rate of peritonitis (27.8% vs. 4.4%, p < 0.01) and the need for emergent surgery (36.1% vs. 11.1%, p < 0.01) was noted in patients with COVID-19 who required hospitalization for treatment. A trend toward a higher rate of perforation (50.0% vs. 31.3%, p = 0.11), abscess formation (38.9% vs. 35.6%, p = 0.82), and fistula formation (8.3% vs. 2.2%, p = 0.32) was also noted; however, these did not reach significance. Detailed results are available in Table 1.

Comparison of Complication Rate by Time of Acute Diverticulitis Diagnosis

As detailed in Table 2, 34 patients presented with abdominal complaints and were diagnosed with acute diverticulitis at the same time as COVID-19 infection. Patients diagnosed with acute diverticulitis and COVID-19 simultaneously were more likely to be hospitalized (73.5% vs. 23.4%, p < 0.01) and had longer hospital length of stay (9.5 vs. 15.0 days, p = 0.02). Additionally, they were more likely to require intubation (11.8% vs. 0.0%, p = 0.03), NIPPV (26.5% vs. 8.5%, p = 0.04), or high-flow nasal cannula (38.2% vs. 6.4%, p < 0.01). In terms of acute diverticulitis, a trend toward a higher incidence of acute complicated diverticulitis (67.6% vs. 53.2%) was noted. However, this did not reach significance (p = 0.25). Regarding individual complications, patients who were diagnosed with acute diverticulitis at the time of COVID-19 diagnosis had a higher incidence of intestinal perforation (55.9% vs. 27.7%, p = 0.01), peritonitis (29.4% vs. 4.3%, p < 0.01), and the need for emergent surgical intervention (38.2% vs. 10.6%, p < 0.01). No difference was noted in the incidence of abscess (38.2% vs. 36.2%, p = 1.00) or fistula formation (5.9% vs. 4.3%, p = 1.00).

## Discussion

This study investigated the complication rate of first-time acute diverticulitis in patients diagnosed with COVID-19 between January 2020 and August 2022. Patients presented with varying complaints leading to RT-PCR-confirmed COVID-19 diagnosis, with 44.4% of patients requiring hospitalization due to symptom severity. Of the patients hospitalized for COVID-19, close to 40.7% required escalation of oxygen therapy beyond the use of a nasal cannula, with the average hospitalization length being close to 11 days. Comparing COVID-19 vaccinated versus unvaccinated patients, unvaccinated patients were significantly more likely to require hospitalization (56.1% vs. 32.5%) and advanced oxygen therapy (65.9% vs. 40.7%). The median duration between the diagnosis of COVID-19 and acute diverticulitis was 13 days, with 40% of patients found to have acute diverticulitis at the time of their initial COVID-19 diagnosis. Although COVID-19 vaccinated versus unvaccinated patients did not differ in this regard, patients requiring hospitalization for COVID-19 were more likely to have acute diverticulitis at their initial presentation to the emergency department (69.4% vs. 17.8%).

Approximately 60% of patients were diagnosed with acute complicated diverticulitis upon diagnosis, with the most common overall complications being intestinal perforation (39.5%) and abscess formation (37%). Furthermore, a substantial number of patients required emergent surgical intervention for acute diverticulitis (22.2%), likely reflecting the higher perforation incidence. When comparing COVID-19 vaccinated versus unvaccinated patients, a trend toward increased all-cause complications was noted. Although not significant, this trend was mainly driven by an elevated incidence of intestinal perforation (43.9% vs. 35.0%) and peritonitis (19.5% vs. 10.0%). In patients whose COVID-19 infection severity warranted hospitalization, 69.4% were diagnosed with acute diverticulitis at presentation. While a non-significant trend toward an elevated incidence of all-cause complications was noted (66.7% vs. 53.5%), a significantly elevated rate of peritonitis (27.8% vs. 4.4%) and need for emergent surgery (36.1% vs. 11.1%) was noted. A subanalysis of patients was completed for patients diagnosed with acute diverticulitis at the time of COVID-19 diagnosis. Most of these patients required hospitalization for COVID-19 (73.5% vs. 23.4%) and advanced oxygen therapy (76.5% vs. 14.9%), likely reflecting more severe COVID-19 infection. Regarding complications of acute diverticulitis, patients who were diagnosed with acute diverticulitis at the time of COVID-19 diagnosis had a trend toward elevated all-cause complications (67.6% vs. 53.2%), driven mainly by significantly elevated rates of intestinal perforation (55.9% vs. 27.7%) and peritonitis (29.4% vs. 4.3%), with 38% of patients requiring emergent surgical intervention.

The above results suggest a high complication rate in COVID-19-positive patients who develop acute diverticulitis. The noted 59% overall incidence rate of acute complicated diverticulitis in patients diagnosed with COVID-19 represents a marked increase above the published overall incidence rate of 9-25%; however, this should be taken in the context of a more ill patient population in the acute and immediate post-acute infectious setting [14,15]. When examining the literature-reported incidence of individual complications of acute diverticulitis (abscess formation: 16-17%, intestinal perforation: 10%, peritonitis: 1-2%), patients who developed acute complicated diverticulitis within 30 days of COVID-19 infection were noted to have an elevated incidence (abscess formation: 37.0%, intestinal perforation: 39.5%, peritonitis: 14.8%), with intestinal perforation and abscess formation being the most common [14,16,17]. On subanalysis, the overall complication rate was found to be further elevated in patients with severe COVID-19 infection (indicated by the need for hospitalization) or those diagnosed with acute diverticulitis at the time of COVID-19 infection. In the two aforementioned groups, the incidence of intestinal perforation and peritonitis is of particular concern, especially when noting the elevated incidence of emergent surgical intervention. While one cannot directly compare the incidence of acute complicated diverticulitis in acute and post-acute COVID-19 patients to literature-reported rates, the current data suggest that patients diagnosed with COVID-19 who develop acute diverticulitis have a high risk of developing complications, in particular, intestinal perforation and peritonitis. Furthermore, our data indicate that patients with severe COVID-19 or those who are diagnosed with acute diverticulitis at the time of COVID-19 diagnosis are at a particularly high risk of complications.

COVID-19 infection has come to be associated with multiple organ dysfunction. This is thought to be secondary to the production of an acute inflammatory state [18]. While most commonly manifesting as a respiratory disease, there has been increased recognition of COVID-19-related gastrointestinal manifestations [1-3]. While the pathophysiology of acute diverticulitis in itself is currently incompletely understood, longstanding theories postulate that obstruction of intestinal diverticula by fecal matter results in low-grade inflammation and mucosal abrasion, subsequently leading to bacterial overgrowth and microperforation [6]. However, more recent studies have demonstrated an association between chronic inflammatory states, altered intestinal microbiome, and the development of acute diverticulitis [7,8]. As such, it is possible that the generalized inflammatory state associated with COVID-19 infection could predispose, and potentially worsen, the development of acute diverticulitis through inflammation-mediated tissue damage. Indeed, histopathological evidence has demonstrated that COVID-19 infection is associated with the infiltration of lymphocytes and plasma cells into the intestinal lamina propria, resulting in diffuse submucosal endothelial inflammation, microvascular injury, and mesenteric ischemia [4]. Furthermore, the COVID-19-induced intestinal inflammatory cell infiltrate has also been associated with substantial intestinal edema [19]. As such, it is conceivable that the intestinal inflammation and edema may predispose to obstruction of pre-existing intestinal diverticula, facilitating bacterial overgrowth and potentially precipitating acute diverticulitis. Furthermore, the combination of COVID-19-associated microvascular injury and mesenteric ischemia may represent the underlying pathomechanism contributing to the increased rate of diverticular perforation and peritonitis noted in the present study. As such, it seems that not only do COVID-19-infected patients who develop acute diverticulitis have a high overall incidence of complications, they are more likely to develop more severe complications of intestinal perforation and peritonitis.

Our study has several limitations that warrant discussion. First, our study used a retrospective approach to investigate the disease course of acute diverticulitis in patients who were diagnosed with COVID-19 in the 30 days prior at a singular institution. Although the current methodology allows for detailed review within one health system with consistent documentation, patient numbers are limited. Furthermore, our study population consisted of mostly Caucasian individuals with a median age of over 65. As such, our results must be interpreted in the setting of this older population with more comorbidities. Nevertheless, comorbid conditions and demographic data did not differ significantly between our investigated groups (vaccinated versus unvaccinated, hospitalized versus non-hospitalized, diagnosis at versus after COVID-19 diagnosis). Furthermore, given that the mean age at presentation for diverticulitis is often above 60 years, our data remain applicable to the at-risk population. To address the current limitations, future studies should seek to include multi-institutional data or leverage available data from national databases for further investigation, as this would allow for a larger and more heterogeneous population. Furthermore, it would be of interest for future studies to investigate if ethnic or socioeconomic differences exist in clinical outcomes for patients with COVID-19 who develop acute diverticulitis.

## Conclusions

Using a retrospective approach at a large tertiary care academic institution, this study found that patients diagnosed with first-time acute diverticulitis within 30 days of COVID-19 infection had an all-cause complication rate of nearly 60%, with the most common complication being intestinal perforation. Although a trend toward an elevated incidence of complicated diverticulitis was noted in COVID-19 unvaccinated patients, this was not found to be significantly different from COVID-19 vaccinated patients. In patients with severe COVID-19 infection or those who were found to have acute diverticulitis upon initial COVID-19 diagnosis, an elevated incidence of all-cause complications was noted, in particular, intestinal perforation and peritonitis. These findings have potential clinical implications for hospital and primary care physicians faced with COVID-19-positive patients who develop acute diverticulitis during or shortly after infection. Given the high incidence of abscess formation, intestinal perforation, and peritonitis in this patient group, patients may benefit from increased clinician vigilance and monitoring for the development of complications.
